# Dr. George Reed

**Published:** 1912-05-25

**Authors:** 


Dr. George Reed, M.D. St. Andrews, late physician to the Salford Royal Hospital, has died at his residence near Newhaven. The greater part of his medical life was passed in hospitals, and he had an extensive administrative experience. After qualifying from St. Bartholomew's Hospital in 1858, Dr. Reed served for a time as house surgeon at his own hospital and proceeded to the post of resident medical officer at the London Fever Hospital, Islington, where in those days an exceptionally fine experience of typhus and typhoid fevers was to be obtained. Later, he also held the appointment of medical superintendent of the Manchester Royal Infirmary, where he had ample scope for his administrative ability. Eventually settling in practice, he did not lose touch with the hospital world, but became later one of the physicians to the Salford Royal Hospital.

